# Why Forensic Pathologists Maintain Uncertainty in Reporting Causes of Death: How Communicative Uncertainty Devices Shape Reasoning

**DOI:** 10.1111/1467-9566.70105

**Published:** 2025-10-13

**Authors:** Mikaela Sundberg

**Affiliations:** ^1^ Department of Sociology and Stockholm Centre for Organizational Research Stockholm University Stockholm Sweden

**Keywords:** communication, forensic medicine, reasoning, uncertainty, uncertainty work

## Abstract

This paper examines how forensic pathologists in Sweden understand and reason around how to communicate the un/certainty of cause of death, given their position to other actors. The paper explores uncertainty work related to the understanding and reasoning on how to use a degree of certainty scale. To define the scale, I introduce the concept of communicative uncertainty device, a concept which can stimulate new questions and comparisons across domains. I outline conservative and liberal approaches to reporting certainty and how dependency on the police for circumstantial information maintains communication of uncertainty. The risk of being called to court gives careful formulation of un/certainty weight, but collegial reviewing shapes more precisely what to write.

## 1 Introduction

Understanding causes of death and patterns of them in the population is part of public health work whereas deeper understanding of individual cases of death can be important for treatment evaluations, legal issues and for the bereaved. However, cause of death is not always obvious, and this is especially the case during forensic pathological examinations of suspicious, sudden or unexpected death. How do forensic pathologists reason around how to report un/certainty regarding their conclusions about causes of death?

The medical literature on communication of diagnostic uncertainty is voluminous (see e.g., Cox et al. 2021; Medendorp et al. 2021) and practitioners' hesitance to communicate uncertainties regarding medical conditions, treatment plans and predictions to patients is well‐established (Braddock et al. 1999; A. L. Simpkin and Armstrong 2019). In contrast to the uncertainties of the future at the centre of most medical work, forensic autopsies deal with uncertainties of the past (cf. J. G. March and Olsen 1975), which are reported to referring authorities. Communication of medical knowledge to such audiences is much less explored compared to communication with patients (cf. Boyd 1998; Dieudonné et al. 2024).

The work and organisation of forensic medicine exhibit a great deal of variation across countries, and also within the United States and United Kingdom. The legal authority requesting forensic autopsies can be a coroner, a medical examiner, the magistrate, the police or the procurator fiscal. The professional status of the medical examiners (i.e., as a medical speciality or not) performing forensic autopsies and their affiliations (i.e., to an independent office, public agency or private practitioner) differ. Previous research studies show how professional positioning and interactions influence the content and form of forensic death investigations. Timmermans' (2005) ethnography of death investigations in a forensic office in the United States demonstrated how forensic medical examiners cooperate closely with the police and other legal professions. Medical examiners risk losing their credibility by delivering false positive reports on suicides, making them conservative in determining suicide as a way to protect their professional autonomy to classify and explain suspicious death. Leslie (2016), in turn, showed how the collegial deference of medically trained coroners in a chief coroner office in Ontario, Canada shaped the content of death certificates, expurgating them when investigating the quality of care delivered by professional colleagues (see also Timmermans 2005). Drawing on insights on how the organisation of forensic death examination affect work, this paper examines how forensic pathologists in Sweden reason around how to communicate the un/certainty of cause of death, given their position in the Swedish institutional context.

In Sweden, the National Board of Forensic Medicine (RMV, short for the Swedish name *Rättsmedicinalverket*), a government authority, employs all physicians specialised in forensic pathology and resident physicians training to become specialists who perform autopsies. The Swedish centralised system of forensic medicine thus contrasts with previous studies of coroner‐based systems (e.g., Jones 2018; Leslie 2016; Prior 1987), the medical‐examiner based system studied in the United States (Timmermans 2005, 2006) and the situation in France, where the magistrate requests forensic autopsies (Juston 2018). Studying Sweden therefore adds to the understanding of the various ways in which medical specialisations work across different countries (see e.g., Weisz 2006).

About 90,000 people die per year in Sweden, and about 5500–6000 of those go through forensic autopsy, which is a comparatively high rate in international perspective. According to the Autopsy Act (SFS 1995:832), physicians who establish death are obliged to contact the police if external factors (i.e., homicide, suicide, accident or malpractice in health) leading to death cannot be excluded or if there is a need to establish the identity of the individual. The police decide whether to request a forensic post mortem examination on these grounds and close a great majority of cases upon receiving the autopsy report because few of them indicate any crime. In 2024, 50% of all cases reported by RMV were classified as disease‐related (i.e., natural), 22% as suicides, 21% as accidents, 2% as caused by another person (homicide) and in less than 5% the intention of the dead (whether death was due to accident or suicide) was unclear (National Board of Forensic Medicine 2024). These statistics refer to conclusions about *manner of death*—the circumstances of death which are most crucial for determining subsequent police work. In this paper, I explore the reporting of *cause of death*, referring to the anatomical and physiological hence more medical, aspects of death. The Swedish system renders it suitable for studying the *epistemic* aspects of forensic medical work because the extensive referral of cases makes the forensic pathologists examine a great variety of causes of death. This forensic medical work is not by definition part of a legal inquest the way it is in coronial systems (cf. Ryan et al. 2025).

Forensic pathologists in Sweden are required to formulate conclusions about cause of death in their autopsy reports in terms of if findings and circumstances ‘show’, ‘speak strongly for’, ‘speak for’, ‘possibly speak’ for a specific cause of death, or whether conclusions cannot be drawn. This so‐called *degree of certainty scale* contrasts with what Timmermans (2006) demonstrated on un/certainty communication. Timmermans (2006, 67) asserted that whereas the tone of scene investigation reports was indefinite, the autopsy report synopsis stated cause of death (i.e., cause of death: myocardial infarction) with unwarranted certainty. However, the certainty by the end of death investigation was not necessarily expected to last. Timmermans (2006, 114, 119) argued that the risk of having the results dissected and contested by lawyers and consulted counter experts during court testimony sets a high professional standard for how forensic pathologists gather and handle evidence for all death determinations, even if few leads to trials. During homicide trials, counselling‐attempts to raise uncertainties around any aspect that could serve the interests of the defendant are anticipated. However the effects of potential scrutiny in court proceedings in systems less adversarial than in the United States, such as in Sweden, is unknown. How forensic pathologists in such systems formulate their knowledge claims on specific causes of death, prior to knowing if and how their reports will be used in courts of law, is thus unknown. Although court proceedings are beyond the scope of this article, what legal actors can expect and hope for regarding the certainty of knowledge claims about death is nevertheless at stake here. This may influence the weight of autopsy reports in court (see Cramer et al. 2009). However, more explicitly, the article concerns the credibility and stability of forensic pathological sources for making knowledge claims about death in the first place, and the meaning and role of un/certainty in relation to the possibilities to communicating them.

## 2 Uncertainty Management, Uncertainty Work and Communicative Uncertainty Devices

Uncertainty of medical knowledge is a major field of research, and a great deal of sociological studies on the topic focuses on how to manage it (Mackintosh and Armstrong 2020). In her classic work on medical uncertainty, Fox (1957) claimed that medical knowledge is inherently uncertain because of all of the gaps and unknowns and because the amount of medical facts is ever expanding and impossible to completely master. Gaining medical confidence by learning to manage the limitations of medicine is hence a fundamental part of socialisation for the aspiring doctor (Fox 1957). Building on Fox, Light (1979) argued that successful socialisation involved sufficient control to make decisions, tempered by the acknowledgement of uncertainties. Timmermans and Angell (2001, 355) questioned the view of uncertainty as ‘a personal condition moving from one polar extreme (uncertainty) to the other (control) instead of as a process of gaining expertise’. In other words, physicians need to manage and ‘tame’ (Pickersgill 2020) the uncertainty of medical knowledge continuously throughout their careers. If certainty is ‘a moral ideal to be achieved’ (Adamson 1997, 135), uncertainty becomes a failure (cf. A. Simpkin and Schwartzstein 2016). Reluctance to communicate uncertainty is then unsurprising. However, if seen as two forms of modus operandi in relation to practical reasoning, on the one hand, and theoretical discourse, on the other, certainty and uncertainty are not necessarily mutually exclusive (Atkinson 1984).

Shifting the perspective from the purview of how individual physicians experience or deal with uncertainties, other scholars have explored uncertainty as a collective phenomenon. Moreira (2011, 1335) defines uncertainty as ‘the non‐determinate or unsettled quality of a statement or knowledge claim’. The reference to ‘unsettled’ points at the collective character of uncertainty (van Loon and Bal 2014, 45). Uncertainty is not a given quality: ‘Work is required to reveal uncertainties in knowledge claims, or equally to keep them closed’, Moreira (2011, 1335) argues. Moreira (2011, see also Moreira et al. 2009) explored public controversies around diagnostic conventions and drug rationing, demonstrating how uncertainty can be mobilised and generative for knowledge production, and not necessarily detrimental for it. Forensic medical death investigation is a form of limited knowledge production process generating specific claims about unique cases of death (cf. Cole 2013). Exploring how to express the un/certainty of such results, my concern is the limited and mundane type of un/certainty work that takes place in local knowledge production processes (cf. Pickersgill 2011). In the context of Swedish forensic death examinations, the degree of certainty scale is central for understanding this, and I define it as a communicative uncertainty device to do so.

Communicative uncertainty devices express uncertainty of knowledge claims and they exist in manifold kinds. Singular, casual hedge phrasing (e.g., insertion of ‘probably’ and ‘likely’) is frequent in everyday speech claims. My interest lies in the communication of un/certainty of claims based on knowledge‐producing activities, including, for example, medical examinations, investigations or analysis of some sort. However, the communicative devices used here can be fairly random too. Research studies show radiologists' (e.g., Callen et al. 2020; Shinagare et al. 2019) and clinical pathologists' (e.g., Gibson et al. 2022; Lindley et al. 2014) unsystematic hedge phrasing in reporting results to referring physicians. In contrast, several universally established concepts express the probability of statistical results. The confidence interval expresses the certainty of estimates for a statistical parameter, whereas the *p* value is the probability that the observed effect within the study would have occurred by chance, rather than reflecting an effect in reality. As these examples show, the form of communicative uncertainty devices can be qualitative or quantitative, disorganised or systematic. Their use can be local or universal, spontaneous or obligatory. Devices can also be expressed in combinations, such as when physicians present a mix of probabilistic outcomes to patients (Cortez and Halpin 2020). Regardless of characteristics, the understanding or use of a specific device is never given. Universal use of standardised devices requires local adaptations (Timmermans and Berg 1995).

Studying a domain similar to forensic pathology in terms of position relative to actors of the court, Kruse (2013) analysed how the Swedish National Forensic Centre expresses the uncertainty of forensic analysis of traces from crime scenes. By inferring the statistical probability of a hypothesis given prior evidence, uncertainty is expressed as a likelihood ratio in the form of the strength of one proposition over the other (e.g., whether the fibres found on the crime scene derive from the sweater of the defendant (Kruse 2013)). In practice, the likelihood approach requires a great deal of tinkering and estimations, rather than calculations (Kruse 2013, 663–665). In other cases, the levels, degrees or expressions of communicative uncertainty devices may reflect customs about how to express un/certainty, instead of estimations or evaluations. By exploring the reasoning around the degree of certainty scale as a specific communicative uncertainty device, I demonstrate the usefulness of the concept for studying uncertainty work and contribute to the growing work on medical uncertainty as a collective, rather than exclusively individual affair.

## 3 Methods and Data

This paper is based on findings from a research project on cause of death knowledge production in general, and how data on causes of death, as a trajectory based on the interconnected practices involved in examining, determining, reporting and registering causes of death, is generated in particular. The research project is hence studying cause of death knowledge with an eye to the production chain of statistics, meaning that I do not explore whether or not death is crime‐related. Potential legal processes including court proceedings, are thus beyond the scope of my research at the project‐level.

To capture the reasoning around how to communicate causes of death, the present analysis is based on semi‐structured interviews with forensic pathologists, and several informal conversations with forensic pathologists during visits to forensic pathological units. Data collection also includes observations of forensic autopsies, but they reveal little of the examiners' sense making when formulating conclusions later, once examinations and tests are complete. Therefore, they are not used for the present analysis.

At the time of the study (2023–4), almost 70 specialists and residents in total worked with cause of death investigations in the six forensic medical units of the RMV. The smallest office had seven physicians and the largest 17. I conducted between three to five interviews in each unit, as a means to maximise variation for the purpose of a richer material and analysis. The study followed all necessary ethical procedures required by Swedish Law.

For this paper, I draw upon 24 interviews in total. Nineteen interviewees were forensic pathological specialists, most of them with experience also from other areas of medical practice (e.g., emergency, health care centres). Some had an additional speciality licence (e.g., clinical pathology, radiology), typically acquired prior to forensic medicine, whereas three had left RMV and shifted to different areas of medicine. Specialists were generally more talkative, speaking more freely, and expressing more nuanced views compared to residents, who are also considerably fewer. For these reasons, I prioritised specialists and interviewed only five residents in total. All interviews began with providing oral informed consent and lasted between one and 2 hours—most of them were 1.5 h or more—and were recorded and transcribed verbatim. The interview guide included questions about, among other things, the last post mortem conducted and the last case closed, probing into as much details about concrete work as possible.

The importance given to how to express un/certainty struck my attention early on. The present article builds primarily, not exclusively, on the probing of examiners' reasoning around when to express certainty. I use the understanding of the expression ‘show’ as my main entry point here. This is because it is the only expression by which forensic pathologists in Sweden claim certainty, making their deliberations about communication of certainty versus uncertainty most visible and pointed in relation to it. It is hence, a suitable prism for exploring such matters in a specific yet instructive way, while drawing on other parts of the material to place communication of certainty in relation to audiences.

For the purpose of this paper, I draw upon a sub‐set of my overall coding of the entire material, conducted in N‐VIVO. I sorted all material related to the use of the degree ‘show’ in one code first. Systematically exploring variation revealed differences in how to express certainty, which I refer to as the conservative and liberal approach. I also used material sorted into codes about the use of circumstantial information and relation with the police to develop more in detail how this affected expression of certainty. At several occasions, forensic pathologists spontaneously mentioned appearance in court. Because of its importance in previous research, I explored systematically the context of this talk in my interviews.

The analysis is limited to the interpretation of findings and the understanding of uncertainty communication when writing the autopsy report, based on interviews. I focus on the perspective of the forensic medical examiners relative to their work with producing a result; how and why they express themselves the way they do. Hence, the analysis does not pertain to the frequency of different expressions in the degree of certainty scale relative to different causes of death written in the autopsy reports, how readers (the police, prosecutors, lawyers) use or interpret autopsy reports nor how the scale was developed and introduced.

To protect the anonymity of cited interlocutors, I refer to them as specialists (e.g., S5) or residents (e.g., R2). I have edited details of the cases referred to and translated all quotes from Swedish. I refer to forensic pathologists and medical examiners interchangeably but use the latter primarily when referring to specific autopsies/post mortem examinations.

## 4 The Degree of Certainty Scale as a Communicative Uncertainty Device

This paper explores forensic pathologists' reasoning on how to communicate un/certainty, but a brief introduction to forensic medical examinations is necessary to understand the work preceding that. The forensic medical examination begins with a forensic medical assistant taking photos of the body, moving it to a bench and sampling body liquids for toxicological analysis. The forensic pathologist carefully examines the body externally, listing and measuring any lesions or scars, noting the colour of wounds, etc. The assistant then opens the chest cavity and eviscerate the organs for separate inspection. The examiner scrutinises and slices several organs (e.g., liver, kidneys, spleen) to look for tumours or other abnormalities. In many cases, forensic pathologists cut samples of organ tissue and inspect the prepared samples microscopically (i.e., histopathology), looking for infections, tumours etc. that were less visible during the autopsy. How far to pursue the investigation in terms of tests and examinations is generally up to the examiner, but two‐examiner investigations of suspected criminal cases are typically directed further by concerns of the ongoing pre‐trial investigations and the external examination is more extensive. Out of 5851 reported forensic autopsies during 2024, 258 (4%) were two‐examiner autopsies (National Board of Forensic Medicine 2024).

The autopsy report, finalised weeks after the autopsy, is the main product of the forensic death investigation, written for and sent to the police. The report includes background information (i.e., from medical records, relatives and witnesses), description based on the external examination of the body and the internal examination of organs, toxicology results, microscopic examination of tissue samples and if applicable, nonstandard, exceptional tests or examinations (i.e., genetic testing, neuropathology). The reports end with a statement on conclusions about cause of death and the manner of death, which must be expressed using the degree of certainty scale. The end of the autopsy report provides a written definition of each degree (see Table 1).

**TABLE 1:** Degree of certainty scale.

Degree	Definition
Show	Findings are typical and alternatives excluded
Speak strongly for	Findings have typical characteristics, probability of other alternatives are very small
Speak for	Findings have common characteristics; alternatives are possible but less likely
May speak for/possibly speaks for	Findings have characteristics that may occur, alternatives are almost as likely
Do not allow conclusions about	Finding/results have no or unspecific characteristics

The degree of certainty scale is a qualitative, standardised communicative uncertainty device. The same expressions are used for other certificates from RMV, including forensic medical certificates following examinations of injuries on the living and medical age determination in refugee cases (to determine if someone is under 18, and thus a minor). The scale has been used for a long time (see e.g., National Board of Forensic Medicine 2001) and the decree to make it mandatory to use (in 2012) thus appears to have aligned with existing habits.

Forensic pathologists in Sweden take elaboration on and subsequent communication of un/certainty for granted to the extent that they criticise not doing or being unable to do so. For example, one specialist (S16) acting as expert in a forensic medical commission in another Scandinavian country commented that there, ‘they write, in principle, “death is *assumed* to be caused by…” and then…“Assumed”, like what? (…) [T]hey had exactly the same phrasing, *all* of them [the reports], regardless if they were really certain or very uncertain’. The specialist found it ‘really weird’ not to elaborate on the uncertainty of the conclusions for each case. Forensic pathologists' critique of the cause of death certificate, where they are not entitled to do so, further illustrates this view: For all cases of death, a physician must fill in a cause of death certificate and send to the National Board of Health and Welfare for registration. The cause of death certificate requests a chain of terminal and underlying causes plus list of contributing causes, expressed in words and ICD‐codes. Physicians must also select manner of death among a list of categories. Mandatory fields do not permit elaboration of un/certainty, but forensic pathologists may add information in a space for free text, even if they do not know if it makes any difference for registration. Given the mission to provide information for statistics at the popu ath certificates is unsurprising (cf. J. March and Simon (1993 [1958]), 186–8). However, for forensic pathologists certificates ‘hide’ uncertainty.

The critical view on cause of death certificates reflects how rare it is for forensic pathologists to express certainty in autopsy report conclusions. Every interviewed forensic pathologist testifies to the paucity of using the expression ‘show’ when presenting conclusions about the cause of death. Extensive bleedings into the pericardium (the fluid‐filled pouch around the heart), resulting from a broken aorta (aorta aneurysm), is one of few natural causes of death that many of my interlocutors mentioned as possibly leading to using the certainty degree ‘show’ in the final statement of the autopsy report. This expression is more likely, but still uncommon, in specific cases of traumatic death, such as being hit by a train or by a single shot in the head (not several, as the effect of each could be difficult to determine).

The decision what to write is a cognitive exercise on the part of the individual physician and forensic pathologists often talked about the choice between ‘show’ or ‘speak strongly for’ as ‘personal’ and ‘subjective’. Nevertheless, I argue that expressions of certainty (also) reflect *social* conventions about how to do so that forensic pathologists keep alive *collectively*, primarily by collegial reviewing. This shapes the content of writing (what degree of certainty to communicate). Subsequently, I distinguish between conservative and liberal approaches to reporting certainty and present their main differences. Building on this, I draw attention to the pivotal role of possible alternative causes of death and how the pathologists' professional dependency on the police informs their maintenance of uncertainty. Therefore, it is the risk of being called to court that ultimately makes careful formulation of un/certainty important. This is not only due to potential scrutiny by lawyers taking place there, but also a matter of positively distinguishing forensic pathology from other medical specialities.

## 5 Conservative and Liberal Approaches to Communicating Certainty

Collegial reviewing is an integrated aspect of finalising autopsy reports within the Swedish system of forensic medicine. Prior to becoming specialists (which typically takes about 5 years and requires, among other things, to have conducted 400 autopsies), residents' autopsy work is surveyed by specialists, with whom they discuss procedures, tests and findings. Specialists also review the autopsy report of the cases they oversaw. The detail of reviewing depends on the case and the length of experience of the resident, but it is common to double‐check histopathological findings in the microscope, read the entire report and discuss findings and conclusions. Although not as extensively as resident reviewing, specialists also peer‐review reports within the same office. In addition, increased homogenisation is promoted by regular circulation of a few reports from each unit to other units, according to a specific schedule for external reviewing.

By reviewing, specialists learn how colleagues present findings and conclusions and comment upon them. The degree of certainty used in the concluding statement is a typical aspect to remark upon in reviewing, but the author's autonomy on what to write remains. In two‐examiner autopsies, both examiners must agree on what to write in the autopsy report. One examiner (S7) described a disagreement regarding such a case, where there was ‘a murder, when someone had been shot in the head and it was caught on surveillance camera (…) You didn't see that he died, but he collapsed and then ambulance staff came and they established cardiac arrest. Then… one person thought [we should write] “speak strongly for” and one “shows”[regarding cause of death]’. This quote appeared in the context of the examiner talking about being open to using ‘show’, whereas some ‘others’ would, presumably, ‘never’ do so. Drawing such distinctions is common among my interlocutors, and the basis for what I refer to as conservative and liberal approaches to certainty communication.

The conservative approach entails not using the expression ‘show’ in the concluding statement of the autopsy report, but reasons vary. Occasionally, ‘speak strongly’ seemed taken for granted as the highest degree of certainty to use. One recent specialist (S12) pondered upon how she concluded a report on a case where someone had been shot in the head, from the back and forward. Despite telling me that ‘it's absolutely what killed the person’, she did not express it with certainty, but used ‘speak strongly’ to express her conclusion, seemingly used out of habit as the highest possible degree to use. A resident (R1) with only a couple of years of experience reported never having written ‘show’ in concluding an autopsy report: ‘It's so inscribed that you never do it, because you *can* never know’. This indicates socialisation into maintaining uncertainty, justified by certainty as impossible and thus, that the habit was connected to a norm. Challenging norms can be both internally and externally demanding. The following quote is an example.

There’s a twenty‐year‐old with the entire aorta ruptured. What else can it be [than an aorta rupture]? But it’s frowned upon to write “shows”. You want to leave this little, little….I think it’s a tradition (…) But now, when you *know* more, you *can* do more, there’s more research, then I think that one could be *a bit* more, kind of… certain and say, this twenty‐year‐old has an aorta rupture. It can’t be anything else they died of. Still, I write “speak strongly for”.

The specialist (S11) refrained from expressing certainty (i.e., writing ‘show’) about cause of death, not because of the judgement about the cause, but presumably due to the experience of using ‘show’ as socially questionable. Another specialist (S13) expected consequences when going against the ‘tradition’ to avoid ‘show’ (and inspired my terminology of this approach): ‘I know that… I'm perhaps more liberal with higher degrees of certainty… then I know that I need to, to those inevitable discussions that arise, I need good arguments’. Expecting to be to be questioned (unspecified where and by whom) because of expressing certainty manifest the conception of divergent approaches among forensic pathologists.

Even if leaving a bit of uncertainty is the ‘tradition’, it is not necessarily the senior upholding it relative to the more junior. Some of the more liberal said the ‘older’ hesitate to express certainty, whereas others said it is the ‘younger’. For example, one experienced specialist (S16), holding an important position at the unit level, asserted a liberal view: ‘I definitely think there's times when you can use “show”’ and referred to collegial discussions of such cases. ‘The new doctors, in particular’ hesitate to use ‘show’, because they, presumably, claim that ‘you can't exclude *everything*’. The specialist found the view exaggerated. ‘It's maybe… one chance in a million that it’s in another way’, the specialist added, as to imply that this was not a reason to deny certainty.

In the next section, I examine what fundamentally distinguishes the two highest degrees in the certainty scale, that is, when alternative causes of death can be excluded versus when ‘some space’ for alternatives (i.e., using ‘speaks strongly for’) is necessary. I show how this depends on conclusive findings, the role given to imaginable versus specific alternatives, and the inevitable uncertainty of information provided by the police.

## 6 Evoking Alternatives as Uncertainty Work

One source of uncertainty in cause of death determination concerns the chronology of events. A ruptured aorta aneurysm is a definitive cause of death, incompatible with life. A natural cause of death by itself, what if it was a punch in the chest that led to the bleeding—can that be excluded? In other cases, a trauma‐induced cause of death (i.e., external violence, accident) might seem evident, but what appeared prior to the trauma? Did the person have a deadly heart attack before being shot, for example? Car accidents are cases in point. It is often difficult to determine whether a car accident was intended (suicide) or not, and in the latter case, if it was the crash or something triggering the crash that caused death. One specialist (S6) reasoned around the difficulties of excluding alternatives, besides dying from a car crash itself:

With traffic accidents etc., then many of my colleagues use “show”… that the injuries caused death, and they have massive multiple injuries due to crash violence (…) but could something have happened *before* that? Did the person die of ventricular fibrillation *before* the car crashed? (…) Well, that isn’t possible to exclude entirely. (…) Because ventricular fibrillation you can’t see. You can see an acute myocardial infarction, but not immediately after death. You must have some time of survival to see it histologically, in the microscope. But you can suspect it, depending on the *findings* you *see* (…) It’s about probabilities, and it’s not very common that we use “show” and that’s why. We say “speak strongly.” It leaves a *bit* of space for something else.

In my interpretation, the pathologist's reference to ‘many of my colleagues’ points at *their* liberal approach to expressing certainty in car accidents cases, contrasting with *her* (and other colleagues, ‘we’) conservative view, according to which exclusion of alternatives must be based on findings. Findings refer to significant observations during the forensic medical examination, including external examination, examination of organs, tissue samples, toxicological tests and potential supplementary investigations. What is not found and what cannot be explored is of importance because it helps the examiner to exclude potential alternatives (see also Timmermans 2006, 140).

However, precisely *what* ‘something else’ that could have led to an alternative cause of death could be is not necessarily specified. One resident (R5) recalled having expressed certainty about the cause of death in cases with single shots in the head, but asserted not having done so and finding it difficult to imagine doing so for anything disease‐related.

There may be many disease processes that we are unaware of or that we have no idea about how they occur, or if they occur, or that we can’t show in some way. Perhaps it’s strange to put what we can see in relation to what we don’t know if it exists at all, but still, there might be some physiological process that made you tip over [and die].

Reminiscent of inherent uncertainties of medical knowledge (cf. Fox 1957; Atkinson 1984), the resident speaks of what is not known or cannot be seen, implying these as possibly affecting death. Within the conservative approach, such unspecified, unknown possibilities suffice to justify the necessity to express a slight uncertainty. In fact, some forensic pathologists occasionally remarked and joked about how the alternative cause of death that ‘some colleagues’ seem to consider for justifying not using ‘show’ would require ‘God's hand’, ‘beamers from Marsians’ or implausible weapon designs. Talking along those liberal lines, one specialist (S10) asserted that simply imagining alternatives is insufficient.

If you think in bizarre ways, then you can always think that it could have happened in a different way, but you must also be somewhat reasonable…. What does the world look like (laughter) and how do things work? You always have circumstances too. You have a police report on that the person was found on the street, there were empty cores there etc.

The quote suggests that circumstances help in understanding what happened and what did not. However, the role of circumstantial information for excluding alternatives is debatable and pertains to how forensic pathologists rely on police work.^1^

### 6.1 Managing Dependency, Managing Uncertainty

Forensic pathologists often highlight documentation of an event, on a surveillance camera or by witness accounts, as crucial for expressing certainty. For example, if a shooting appears being a homicide, witnesses or surveillance camera can support if death could possibly have occurred prior to the shooting (i.e., through a heart attack). Forensic pathologists also mention multiple massive injuries from train accidents as cases where they express certainty. This requires witnesses to make sure that the person arrived on the tracks alive, and was not placed there, injured or already dead. When asked about expressing certainty about cause of death in autopsy reports, one specialist (S9) with many years of experience answered rarely doing so, but

then you kind of *assume* that incoming information is correct. I mean…it says in the police report that this person has been witnessed walking onto the tracks and being hit by a train. Then I assume that the information is correct; that this person actually did walk onto a track. Then it’s total bodily destruction that is the cause of death. Then there’s no space for anything else.

Reliability of circumstantial information is necessary for excluding alternatives, but circumstantial information can be of different kinds. According to Timmermans (2006, 108, 259), forensic pathologists distinguish between subjective biographical information and objective information such as medical records and biochemical tests, preferring the latter. The question I address here is, what is the role of information, especially non‐medical, derived from sources other than the post mortem examination for expressing certainty?

The police provide all circumstantial information. The autopsy request presents a brief background (a few sentences, sometimes including information on diseases and/or substance abuse). Photos of the body and surroundings are often included. Forensic examiners are not part of the health care system and to read medical records, the examiner must request specific records through the police, and reception relies on them.

With the exception of pre‐trial investigations of suspected homicides, forensic pathologists drive the death investigations themselves. According to a specialist (S16), alleging to frequent requests to the police for more information, ‘The death investigators of the police get very *uninterested* quickly, if you say so. *They* don't want to go back [to home of the dead or place of death] to look for lots of things, they don't think they have the time (…) If it's not a murder or something’. My point is that forensic pathologists do not necessarily receive all the information *they* desire (not to question police priorities) and do not know to what extent relevant circumstantial information is missing. Circumstantial information is beyond their control. Elaborating on the situation in relation to expressing uncertainty, one specialist (S15) asserted:

You must clarify what it is that *I* govern in some way. What *I’m* in control over for the judgement and what’s pending on me and what’s, sort of, entirely… standalone from my… what to say… my investigation. What is it that’s only *given* to me? (…) Everything that can that can change (snapping fingers), that can be taken away from you anytime. If you should be kind of crude (…) It can happen all of a sudden, it’s a piece of information like this and like this. Of course, normally you can kind of trust the information you get, but it’s still good to kind of, draw a boundary *yourself* for what’s my parameters that I can be entirely sure of and what’s the rest in this case, that I receive and which could change.

This description illustrates how forensic pathologists depend on the police and how the specialist considered how for each specific case. However, it is not only a matter of potential missing or unstable information but also about the skill of understanding different types of material. Forensic pathologists are trained to interpret what they have seen in the body with their own eyes, and felt with their fingers. For both these reasons, they are more in charge of what they have found and tested as part of their investigation of the body compared to whatever information the police give them.

The extent to which circumstantial information play a role for cause of death depends on the case (whereas it is generally crucial for determining manner of death). Therefore, the possibility of circumstantial information to change—by being withdrawn, added or revised—is a fundamental reason to maintain at least a bit of uncertainty when presenting conclusions, thereby managing their dependency on the police. It is a way of avoiding to being caught with promoting exclusively what turns out to be wrong or questionable, despite the fact that the conclusions drawn on the basis of the material at hand might well have been ‘right’. A deeper analysis of how forensic pathologists interact with the police regarding circumstantial information is beyond the scope of the present analysis. Notable, however, is that the police may request a complementary report if conclusions contradict police information which the examiner has not received. This is potentially a more collaborative exercise compared to if lawyers (or others) criticise the report or forensic pathologist in court. Timmermans (2006) argued that the risk of being questioned in court shape professional standards of forensic pathologists, and I conclude the empirical analysis by examining how this applies to uncertainty reporting in the Swedish context.

## 7 How Thinking About ‘the Court’ Shapes Uncertainty Work and Bolster Professional Pride

In 2024, forensic pathologists from RMV attended 119 court negotiations; about a third of them concerned death investigations (National Board of Forensic Medicine 2024). In other words, forensic pathologists attend court proceedings dealing with less than 1% of the total number of forensic autopsy cases. If they do, heavy interrogation is unlikely. The Swedish system is considerably less confrontational compared to Anglo–Saxon law (see e.g., Nermark 2018) and because all practicing forensic pathologists are employed by the same government agency, they cannot be pitted against each other in court. One specialist commented upon a relatively recent court appearance during a trial of a debated case. The defence counsel called an external expert (a retired forensic pathologist) for an alternative view on the cause of death. Despite 20 years as a forensic pathologist, my interlocutor (S14) commented: ‘It was a useful experience to sit in court and really argue for oneself’. This implies that such questioning of forensic pathologists is uncommon in Sweden (see also Thiblin and Michard 2014, 22).

Based on the above, one might expect that potential court appearances play a minor role for forensic pathologists in Sweden. This is not entirely the case, however. Reminiscent of how the forensic scientists Bechky (2021) studied referred a great deal to testimonies in court, despite the fact that they rarely appeared there, many of my interlocutors mentioned ‘the court’ (*rätten*) at some point. Such unprompted talk mainly concerned that forensic pathologists must be able to motivate their conclusions about causes of death. Interestingly, it occurred in the context of distinguishing their work from cause of death determination among general practitioners and hospital physicians, who draw primarily on medical records for doing so (see Bloor 1991; McAllum et al. 2005). Forensic pathologists hold themselves as more accountable, because of their extensive work and preparedness to be questioned: ‘Everything boils down to the courtroom situation. *How sure or unsure are you about something*? You can't sit in court babbling and guessing. It must be as substantiated as possible’, one specialist (S3) asserted. Forensic pathologists also differentiate their work from clinical pathologists, who conduct clinical autopsies and report conclusions to the referring physician, who then fills in the cause of death certificate (supposedly uncritical towards the clinical pathologist's conclusions).

*Here* [at RMV], you constantly think: How do I explain this? How do I defend myself is someone questions it? Okay if you don’t do it in great detail for a heart attack, but the way of *thinking* is still that you must be able to explain yourself and kind of “why do you say this and not that?” And when you are talking about this, how strongly do you mean? This is the way we are taught… that everything can be questioned.

The cited examiner (S7) suggests that the need to prepare for questioning depends on the case, using a common type of sudden, natural death, unlikely to lead to legal processing, as an example. The cases leading to trial has typically been classified as ‘high suspicion of crime’ (‘low suspicion of crime’ is the lowest and most common classification) by the police already upon request. This means that extended, two‐examiner autopsies took place and that the risk of being called to witness is evident, but small. Under exceptional circumstances, the police may return to closed, routine cases, examined by a single forensic pathologist, and start homicide investigations. Hence, thorough work on every conclusion is necessary to deal with the slim risk of suddenly and unpredictably ending up in court, being a form of uncertainty management concerning the future for the forensic pathologist. As one resident (R2) asserted, ‘You know that 95% of the cases will never go to court (…) but you don't know which ones [will], so working here, it's better to have the approach that it is possible to go to trial with all cases and explain there. Then you don't have to do in any specific way [when that happens]’.

Reminiscent of Timmermans' (2006) finding, conscientiousness, to the point of being meticulous, is standard for forensic pathologists in Sweden too. Expressing an appropriate degree of certainty is part of this ideal. In the words of one specialist (S10): ‘A really important trait for a forensic pathologist is to always be self‐critical, scrutinising and as conscientious as possible, even in the common cases that doesn't have any legal consequences, that you really make an effort to find the right level of certainty’. Critical self‐examination of how to express un/certainty is a specific form of socialised uncertainty work whereby the forensic pathologist weighs formulations given findings and circumstances. They do so out of fear of interrogation, but it is also a source of professional pride for their speciality relative to other medical professionals.

## 8 Discussion

The aim of this paper has been to show how forensic pathologists deal with and reason around how to communicate un/certainty, exploring how the position in and perspective relative to their institutional ecology affects this. The analysis of different approaches to the communication of certainty (i.e., using the expression ‘show’) revealed how maintenance of uncertainty is sustained by a ‘tradition’ on how to write, connected to notions of inevitable uncertainties of medical knowledge and the difficulty of ruling out alternative explanations. Evoking alternatives is an archetypical form of uncertainty work. In Swedish forensic pathology, this uncertainty work is closely tied to the definition (formulations) of the degrees in the certainty scale. To maintain uncertainty reflects the openness to potentially significant yet unknown or unstable police information. Such openness follows from forensic medical examiners' responsibility to present the results of cause of death investigations, while lacking control over the access to material that could influence them. In contrast to how most physicians may establish dominant relationships with clients to deal with uncertainty (Light 1979) in private, maintaining the uncertainty of knowledge claims is a way for forensic pathologists to manage their dependency on the police, who they also report to. This management entails categorisation (Mackintosh and Armstrong 2020, 8–9), whereby forensic pathologists distinguish what belongs to their domain from what belongs to the police. The paper contributes to existing work on uncertainty communication in health and medicine by providing a more reflexive account on medical professionals' considerations about how to communicate uncertainty to audiences where potential consequences are legal rather than health‐related, and where communication might be publicly scrutinised rather than accepted in private.

The position between medicine and law distinguishes forensic medicine from other medical specialities. Timmermans (2006) argued that the risk of being questioned in court affects how forensic pathologists think about their work, making them meticulous. This paper helped to round out the portrait of the ways in which this takes place. Forensic pathologists in Sweden do not only take elaboration of uncertainty about cause of death for granted, but also take pride in doing so. The (slim) risk of presenting results in court influences the importance forensic pathologists place on communicating, and motivating, how un/certain conclusions about causes of death are. Preparedness for questioning, by colleagues and in court, is considered to be a salient feature of their speciality and institutionalised reviewing, by senior colleagues or peers, a way of socialising into it. The status of forensic medicine as a speciality is low (cf. Norredam and Album 2007) and both Leslie (2016) and Timmermans (2006) described deference to medical professionals in reporting deaths related to medical misconduct. This article contributes to knowledge about how forensic pathologists relate to other medical professionals by showing how they take pride in being the (most) accurate physicians and medical reporters of the past. Although their ability to claim certainty about death is partly related to their dependence on the police, their conscientious about *how* uncertain they are is a way to advocate the relevance of detailed knowledge about death.

The degree of certainty scale is not only used to communicate un/certainty, but I have also used it as an entry point for understanding the meaning of certainty. With a literal understanding of the expression ‘show’ requiring exclusion of every imaginable alternative cause of death, forensic pathologists carry the entire uncertainty of medical knowledge and omnipresent possibility of revised circumstantial information on their shoulders. With such an understanding, expressing certainty is impossible. Communicating certainty is feasible (not necessarily reasonable) if the degree of certainty scale is used to express the interpretation of findings given the state of knowledge and circumstantial information at hand. Reminiscent of Atkinson's (1984) different modus operandi, these approaches to certainty orient towards the inherent uncertainty of medical knowledge (cf. Fox 1957), on the one hand, and to practical limited knowledge‐making on the other.

Studying the understanding and use of communicative uncertainty devices is a locus for exploring how knowledge claims are unsettled or closed among those producing them and how situated meanings of un/certainty arise in relation to both stocks of knowledge and communicative devices. The notion of communicative uncertainty device brings together a number of diverse ways of communicating uncertainty under the same umbrella, instigating new questions for future work on uncertainty communication, within and beyond health and medicine. Studying forensic pathology in the Swedish context enabled scrutiny of a specific communicative device for expressing un/certainty, suggesting how communicative uncertainty devices shape the understanding of certainty. However, the focus on forensic pathologists' current reasoning about the certainty scale is a limitation and further, historical work is needed to cover the origins of its definition and use in the Swedish system. In addition to such work, calls for standardised terminology in reporting the uncertainty of results in different medical fields (Gibson et al. 2022; Shinagare et al. 2019) offer possibilities to study when and how specific and standardised communicative uncertainty devices are designed, enforced and implemented, by whom, for what purposes and with what consequences for, among other things, the credibility *vis à vis* audiences.

## Author Contributions

**Mikaela Sundberg:** conceptualization, funding acquisition, investigation, methodology, project administration, writing – original draft, writing – review and editing.

## Ethics Statement

According to Swedish law (*Lag* [2003:460] *om etikprövning av forskning som avser människor*), no application for ethical approval is required for the research conducted within the research project. Projects on human subjects interrogating what is defined by the law as sensitive areas require ethical approval. Considering the focus on professional practices, perspectives, reasoning and judgements in the project, it does not interrogate into what is defined as sensitive area. Given the focus on perspectives in medical practice, it is worth pointing out that personal health is a sensitive research area according to the law. However, this applies only to the living and not to the dead, where ethical restrictions pertain to the use of bodily material exclusively. Consequently, information about previous health issues on unidentified dead persons does not require ethical permission.

## Consent

The author has nothing to report.

## Conflicts of Interest

The author declares no conflicts of interest.

## Permission to Reproduce Material From Other Sources

The author has nothing to report.

## Data Availability

The data are not publicly available due to privacy and ethical restrictions.

## Endnotes

^1^
For discussion on how such information *should* be used in judicial cases, see for example, SOU 2006:103, 66, Thiblin and Michard (2014, 25).

## References

[shil70105-bib-0001] Adamson, C. 1997. “Existential and Clinical Uncertainty in the Medical Encounter: An Idiographic Account of an Illness Trajectory Defined by Inflammatory Bowel Disease and Avascular Necrosis.” Sociology of Health & Illness 19, no. 2: 133–159. 10.1111/1467-9566.ep10934391.

[shil70105-bib-0002] Atkinson, P. 1984. “Training for Certainty.” Social Science & Medicine 19, no. 9: 949–956. 10.1016/0277-9536(84)90324-1. 6515428

[shil70105-bib-0003] Bechky, B. A. 2021. Blood, Powder, and Residue: How Crime Labs Translate Evidence into Proof. Princeton University Press.

[shil70105-bib-0004] Bloor, M. 1991. “A Minor Office: The Variable and Socially Constructed Character of Death Certification in a Scottish City.” Journal of Health and Social Behavior 32, no. 3: 273–287. 10.2307/2136808. 1940210

[shil70105-bib-0005] Boyd, E. A. 1998. “Bureaucratic Authority in the ‘Company of Equals’: The Interactional Management of Medical Peer Review.” American Sociological Review 63, no. 2: 200–224. 10.2307/2657323.

[shil70105-bib-0006] Braddock, C. H., III , K. A. Edwards , N. M. Hasenberg , T. L. Laidley , and W. Levinson . 1999. “Informed Decision Making in Outpatient Practice: Time to Get Back to Basics.” JAMA 282, no. 24: 2313–2320. 10.1001/jama.282.24.2313. 10612318

[shil70105-bib-0007] Callen, A. L. , S. M. Dupont , A. Price , et al. 2020. “Between Always and Never: Evaluating Uncertainty in Radiology Reports Using Natural Language Processing.” Journal of Digital Imaging 33, no. 5: 1194–1201. 10.1007/s10278-020-00379-1. 32813098 PMC7573053

[shil70105-bib-0008] Cole, S. A. 2013. “Forensic Culture as Epistemic Culture: The Sociology of Forensic Science.” Studies in History and Philosophy of Science Part C: Studies in History and Philosophy of Biological and Biomedical Sciences 44, no. 1: 36–46. 10.1016/j.shpsc.2012.09.003. 23021588

[shil70105-bib-0009] Cortez, D. , and M. Halpin . 2020. “Uncertainty and Certain Death: The Role of Clinical Trials in Terminal Cancer Care.” Sociology of Health & Illness 42, no. 1: 130–144. 10.1111/1467-9566.13059. 31981223 PMC8345924

[shil70105-bib-0010] Cox, C. L. , B. M. Miller , I. Kuhn , and Z. Fritz . 2021. “Diagnostic Uncertainty in Primary Care: What Is Known About Its Communication, and What Are the Associated Ethical Issues?” Family Practice 38, no. 5: 654–668. 10.1093/fampra/cmab023. 33907806 PMC8463813

[shil70105-bib-0011] Cramer, R. J. , S. L. Brodsky , and J. DeCoster . 2009. “Expert Witness Confidence and Juror Personality: Their Impact on Credibility and Persuasion in the Courtroom.” Journal of the American Academy of Psychiatry & the Law Online 37, no. 1: 63–74. PMID: 19297636. 19297636

[shil70105-bib-0012] Dieudonné, M. , P. Castel , and M. Robelet . 2024. “L’Impossible Contrôle Consultatif Entre Pairs. Le Cas Des Relations Entre Médecins Dans Les EHPAD.” Sociologie du Travail 66: 1. 10.4000/sdt.45504.

[shil70105-bib-0013] Fox, R. G. 1957. “Training for Uncertainty.” In The Student‐Physician: Introductory Studies in the Sociology of Medical Education, 207–242. Harvard University Press.

[shil70105-bib-0014] Gibson, B. A. , E. McKinnon , R. C. Bentley , et al. 2022. “Communicating Certainty in Pathology Reports: Interpretation Differences Among Staff Pathologists, Clinicians, and Residents in a Multicenter Study.” Archives of Pathology & Laboratory Medicine 146, no. 7: 886–893. 10.5858/arpa.2020-0761-oa. 34669920

[shil70105-bib-0015] Jones, I. 2018. “‘It’s all About Justice’: Bodies, Balancing Competing Interests, and Suspicious Deaths.” Journal of Law and Society 45, no. 4: 563–588. 10.1111/jols.12130.

[shil70105-bib-0016] Juston, R. 2018. “Devenir Expert, Rester Médecin? Les Effets de la Spécialité Médicale sur L’Exercice de la Médecine Légale.” Sociologie du Travail 60: 3. 10.4000/sdt.2668.

[shil70105-bib-0017] Kruse, C. 2013. “The Bayesian Approach to Forensic Evidence: Evaluating, Communicating, and Distributing Responsibility.” Social Studies of Science 43, no. 5: 657–680. 10.1177/0306312712472572.

[shil70105-bib-0018] Leslie, M. 2016. “‘I Can’t Put That on Paper’. How Medical Professional Values Shape the Content of Death Certificates.” International Journal of Law in Context 12, no. 2: 178–194. 10.1017/s1744552316000045.

[shil70105-bib-0019] Light, D. Jr . 1979. “Uncertainty and Control in Professional Training.” Journal of Health and Social Behavior 20, no. 4: 310–322. 10.2307/2955407. 541485

[shil70105-bib-0020] Lindley, S. W. , E. M. Gillies , and L. A. Hassell . 2014. “Communicating Diagnostic Uncertainty in Surgical Pathology Reports: Disparities Between Sender and Receiver.” Pathology, Research & Practice 210, no. 10: 628–633. 10.1016/j.prp.2014.04.006. 24939143

[shil70105-bib-0021] Mackintosh, N. , and N. Armstrong . 2020. “Understanding and Managing Uncertainty in Health Care: Revisiting and Advancing Sociological Contributions.” Supplement, Sociology of Health & Illness 42, no. S1: X–20. 10.1111/1467-9566.13160. 32757281

[shil70105-bib-0022] March, J. , and H. Simon . 1993 [1958]. Organizations. 2nd ed. Blackwell publishers.

[shil70105-bib-0023] March, J. G. , and J. P. Olsen . 1975. “The Uncertainty of the Past: Organizational Learning Under Ambiguity.” European Journal of Political Research 3, no. 2: 147–171. 10.1111/j.1475-6765.1975.tb00521.x.

[shil70105-bib-0024] McAllum, C. , I. St George , and G. White . 2005. “Death Certification and Doctors’ Dilemmas: A Qualitative Study of GPs’ Perspectives.” British Journal of General Practice 55, no. 518: 677–683. PMID: 16176734. PMC1464060 16176734

[shil70105-bib-0025] Medendorp, N. M. , A. M. Stiggelbout , C. M. Aalfs , P. K. Han , E. M. Smets , and M. A. Hillen . 2021. “A Scoping Review of Practice Recommendations for Clinicians’ Communication of Uncertainty.” Health Expectations 24, no. 4: 1025–1043. 10.1111/hex.13255. 34101951 PMC8369117

[shil70105-bib-0026] Moreira, T. 2011. “Health Care Rationing in an Age of Uncertainty: A Conceptual Model.” Social Science & Medicine 72, no. 8: 1333–1341. 10.1016/j.socscimed.2011.02.026. 21439698

[shil70105-bib-0027] Moreira, T. , C. May , and J. Bond . 2009. “Regulatory Objectivity in Action: Mild Cognitive Impairment and the Collective Production of Uncertainty.” Social Studies of Science 39, no. 5: 665–690. 10.1177/0306312709103481.

[shil70105-bib-0028] National Board of Forensic Medicine . 2001. “Dokumentation Vid Rättsmedicinsk Undersökning (Obduktion).” QB5.9.2001.

[shil70105-bib-0029] National Board of Forensic Medicine . 2024. “Annual Report.” Registration number X25‐90025.

[shil70105-bib-0030] Nermark, C. 2018. En Granskning av Rättsmedicinsk Sakkunnigbevisning Vid Dödligt Våld. Thesis. Faculty of Law, Lund University.

[shil70105-bib-0031] Norredam, M. , and D. Album . 2007. “Prestige and Its Significance for Medical Specialties and Diseases.” Scandinavian Journal of Public Health 35, no. 6: 655–661. 10.1080/14034940701362137. 17852972

[shil70105-bib-0032] Pickersgill, M. 2011. “Ordering Disorder: Knowledge Production and Uncertainty in Neuroscience Research.” Science as Culture 20, no. 1: 71–87. 10.1080/09505431.2010.508086.

[shil70105-bib-0033] Pickersgill, M. 2020. “Uncertainty Work as Ontological Negotiation: Adjudicating Access to Therapy in Clinical Psychology.” Sociology of Health & Illness 42, no. 1: 84–98. 10.1111/1467-9566.13029. 31769615 PMC7496927

[shil70105-bib-0034] Prior, L. 1987. “Policing the Dead: A Sociology of the Mortuary.” Sociology 21, no. 3: 355–376. 10.1177/0038038587021003004.

[shil70105-bib-0035] Ryan, S. , F. Ribenfors , M. Mikulak , and D. Coles . 2025. “Between Epistemic Injustice and Therapeutic Jurisprudence: Coronial Processes Involving Families of Autistic People, People With Learning Disabilities and/or Mental Ill Health.” Sociology of Health & Illness 47, no. 2: e13855. 10.1111/1467-9566.13855. 39555897 PMC11849770

[shil70105-bib-0036] SFS . 1995:832. The Autopsy Act.

[shil70105-bib-0037] Shinagare, A. B. , R. Lacson , G. W. Boland , et al. 2019. “Radiologist Preferences, Agreement, and Variability in Phrases Used to Convey Diagnostic Certainty in Radiology Reports.” Journal of the American College of Radiology 16, no. 4: 458–464. 10.1016/j.jacr.2018.09.052. 30584042

[shil70105-bib-0038] Simpkin, A. , and R. Schwartzstein . 2016. “Tolerating Uncertainty—The Next Medical Revolution?” New England Journal of Medicine 375, no. 18: 18–1715. 10.1056/nejmp1606402. 27806221

[shil70105-bib-0039] Simpkin, A. L. , and K. A. Armstrong . 2019. “Communicating Uncertainty: A Narrative Review and Framework for Future Research.” Journal of General Internal Medicine 34, no. 11: 2586–2591. 10.1007/s11606-019-04860-8. 31197729 PMC6848305

[shil70105-bib-0040] SOU . 2006:103. “Översyn av den Rättsmedicinska Verksamheten. Tillsyn, Rättsliga Rådet och Rättsläkarens Roll.” Slutbetänkande av RMV/SKL utredningen, Official government report.

[shil70105-bib-0041] Thiblin, I. , and J. F. Michard . 2014. Rättsmedicin i Teori och Praktik: En Guide för Läkare och Jurister. Studentlitteratur AB.

[shil70105-bib-0042] Timmermans, S. 2005. “Suicide Determination and the Professional Authority of Medical Examiners.” American Sociological Review 70, no. 2: 311–333. 10.1177/000312240507000206.

[shil70105-bib-0043] Timmermans, S. 2006. Postmortem: How Medical Examiners Explain Suspicious Deaths. University of Chicago Press.

[shil70105-bib-0044] Timmermans, S. , and A. Angell . 2001. “Evidence‐Based Medicine, Clinical Uncertainty, and Learning to Doctor.” Journal of Health and Social Behavior 42, no. 4: 342–359. 10.2307/3090183. 11831136

[shil70105-bib-0045] Timmermans, S. , and M. Berg . 1995. “Standardization in Action: Achieving Local Universality Through Medical Protocols.” Social Studies of Science 27, no. 2: 273–305. 10.1177/030631297027002003.

[shil70105-bib-0046] van Loon, E. , and R. Bal . 2014. “Uncertainty and the Development of Evidence‐Based Guidelines.” Valuation Studies 2, no. 1: 43–64. 10.3384/vs.2001-5992.142143.

[shil70105-bib-0047] Weisz, G. 2006. Divide and Conquer: A Comparative History of Medical Specialization. Oxford University Press.

